# Harvesting Effects, Recovery Mechanisms, and Management Strategies for a Long-Lived and Structural Precious Coral

**DOI:** 10.1371/journal.pone.0117250

**Published:** 2015-02-23

**Authors:** Ignasi Montero-Serra, Cristina Linares, Marina García, Francesca Pancaldi, Maša Frleta-Valić, Jean-Baptiste Ledoux, Frederic Zuberer, Djamel Merad, Pierre Drap, Joaquim Garrabou

**Affiliations:** 1 Departament d’Ecologia, Universitat de Barcelona, Barcelona, Spain; 2 Institut de Ciències del Mar (ICM-CSIC), Barcelona, Spain; 3 Institut Pytheas, UMS 3470, Station Marine d'Endoume, Marseille, France; 4 LSIS UMR CNRS 7296, Centre National de la Recherche Scientifique, Marseille, France; 5 CIMAR /CIIMAR Centro Interdisciplinar de Investigação Marinha e Ambiental, Universidade do Porto, Porto, Portugal; 6 UM110, CNRS/INSU, IRD, Aix-Marseille Université, Université du Sud Toulon Var, Mediterranean Institute of Oceanography (MIO), Marseille, France; Biodiversity Research Center, Academia Sinica, TAIWAN

## Abstract

Overexploitation is a major threat for the integrity of marine ecosystems. Understanding the ecological consequences of different extractive practices and the mechanisms underlying the recovery of populations is essential to ensure sustainable management plans. Precious corals are long-lived structural invertebrates, historically overfished, and their conservation is currently a worldwide concern. However, the processes underlying their recovery are poorly known. Here, we examined harvesting effects and recovery mechanisms of red coral *Corallium rubrum* by analyzing long-term photographic series taken on two populations that were harvested. We compared the relative importance of reproduction and re-growth as drivers of resilience. Harvesting heavily impacted coral populations causing large decreases in biomass and strong size-class distribution shifts towards populations dominated by small colonies. At the end of the study (after 4 and 7 years) only partial recovery was observed. The observed general pattern of low recruitment and high mortality of new recruits demonstrated limited effects of reproduction on population recovery. Adversely, low mortality of partially harvested adults and a large proportion of colonies showing new branches highlighted the importance of re-growth in the recovery process. The demographic projections obtained through stochastic models confirmed that the recovery rates of *C. rubrum* can be strongly modulated depending on harvesting procedures. Thus, leaving the basal section of the colonies when harvesting to avoid total mortality largely enhances the resilience of *C. rubrum* populations and quickens their recovery. On the other hand, the high survival of harvested colonies and the significant biomass reduction indicated that abundance may not be an adequate metric to assess the conservation status of clonal organisms because it can underestimate harvesting effects. This study highlights the unsustainability of current harvesting practices of *C. rubrum* and provides urgently needed data to improve management practices that are still largely based on untested assumptions.

## Introduction

Overfishing is a major threat to the integrity of world's marine ecosystems. Historical exploitation of marine resources has resulted in drastic population declines, species extinctions and the general simplification of marine food webs [1], [2]. This structural impoverishment has also hindered the resilience of marine populations, lowering their ability to recover after recurrent natural and human disturbances [3], [4]. Understanding the ecological consequences of different extractive practices on the structure and function of populations, especially of those organisms exhibiting a long life span and slow dynamics, is therefore one of the greatest challenges in conservation biology [5].

Precious corals have been harvested and traded worldwide since ancient times due to the high economic value of their carbonate axial skeleton [6], [7], [8]. These sessile invertebrates are considered habitat forming species with a keystone role on coastal systems because they provide structural complexity and host high levels of biodiversity [9]. Additionally, they can enhance fisheries by providing shelter during early stages for some commercially important species of rock fishes, shrimps and crabs [10]. The red coral *Corallium rubrum* is a precious octocoral endemic to the Mediterranean rocky bottoms and adjacent Atlantic waters. Available data showed recent large declines in the Mediterranean yields, suggesting that this fishery is unsustainably managed following boom and bust cycles [11], [7]. Intensive harvesting has resulted in significant shifts in the size structure of current *C*. *rubrum* populations [12], [7], causing a decrease in biomass and mean and maximum colony size [13], [14]. Moreover, current warming and acidification trends associated to global change are major threats for shallow red coral populations [15], [16], [17], [18]. Consequently, there is a growing concern for the conservation of *C*. *rubrum* and the rest of the precious corals worldwide as evidenced by the recent struggle to include the Genus *Corallium* in the Appendix II of Cites [7], [19], [20], [21].

The need for a responsible fishery for *C*. *rubrum* and the negative effects of current practices are widely accepted [11], [21], [22]. However, given the fragility and vulnerability to different threats of this species, all studies addressing harvesting effects and recovery have been carried out by comparing size-class distribution of different populations and periods. [11], [23], [24], [25]. To date, biomass reduction has been only quantified once after a poaching event [26]. Beyond the reported shifts in size structures of *C*. *rubrum* populations [11], [24], sound field and experimental data on the effects of different extractive practices and the recovery process are essentially lacking in the literature.

Due to their low population dynamics and limited dispersal capacities [27], [28], precious corals are considered to have low resilience to disturbances that cause high adult mortality such as harvesting. Paradoxically, *C*. *rubrum* has not undergone extinction despite being exposed to intensive harvesting since ancient times. In fact, the biological processes underlying coral populations' persistence are largely unknown. The early maturity of *C*. *rubrum* (2.4 cm in height) corresponding to an age of 6–10 yr [29, 30], could partially explain this persistence. It allows the colonies to contribute to reproductive output before they reach the size of interest for harvesters. On the other hand, colonial organisms have the capacity to recover after events of partial mortality (e.g. breakage of a branch). Indeed, red coral colonies with signs of breakage and recent re-growth of new branches have been observed [29], [31]. The resilience to harvesting will therefore depend on two main mechanisms: (1) re-growth or clonal growth of colonies suffering partial mortality (i. e. when leaving the basal section) and (2) recruitment by sexual or asexual reproduction (i. e. when eradicating the whole colony). Yet, there is no assessment on the relative contribution to recovery of these two mechanisms from a long-term perspective. This assessment would provide a scientific basis for new management measures that enhance the sustainability of this natural resource.

In this study, we examined harvesting effects and the recovery processes of *C*. *rubrum* using 5–7 years photographic series on two populations located on the coast of Marseilles (France, NW Mediterranean). Fishermen unexpectedly harvested two *C*. *rubrum* populations that were already being studied by our research team and this represented a unique opportunity to establish before / after comparisons as well as to explore directly the recovery mechanisms by following individually affected as well as unaffected *C*. *rubrum* colonies. Our overall goal was to elucidate the demographic process underlying the recovery of *C*. *rubrum* after harvesting events with a particular focus on the relative contribution of re-growth of harvested colonies and reproduction. More precisely we quantified (1) survival and re-growth rates of harvested colonies, (2) survival of non-affected colonies and (3) recruitment rates and survival of recruits. Based on these data, we developed biomass projections to compare population trajectories under different harvesting practices. We contend that the results provide meaningful insights to inform new legislation measures to promote resilience and ensure the conservation of this threatened species and other precious corals also at deeper depths, where most of fishing efforts are concentrated and this type of data is more difficult to obtain.

## Methods

### 1 Study Area

We monitored two Mediterranean red coral populations located along the rocky coast of Massif des Calanques in the SE of Marseilles, France. Due to the specific habitat characteristics of the area, such as submerged cavities and overhangs, along with instability of the water column during summer [32], the development of red coral populations at shallow depths (15–22m) is favored. The studied populations are located in a vertical wall at Riou Island (43° 10' 23.47" N i 5° 23' 13.24" E) (hereafter Riou) and in a cave-like tunnel at Maire Island (43°12'32.34"N; 5°20' 14.01"E) (hereafter Maire).

### 2 Coral Population Monitoring

In each population we monitored 30 quadrants (20 x 20 cm) by setting up 2 permanent plots using PVC screws fixed to holes in the rocky substratum. Each plot was variable in length, depending on the complexity of the substratum, and 40 cm wide. In each sampling, a cord was deployed between the screws and quadrats assembled with a scale were sequentially positioned and photographed above and below the cord throughout the length of each transect. Two photographs from each quadrat (using 2 slightly different angles, ~30°) were used for analysis with photogrammetric techniques which allowed measuring the height of colonies [33]. Transects were photographed using a NIKON D70 with a housing and 2 electronic strobes.

**Fig 1:**
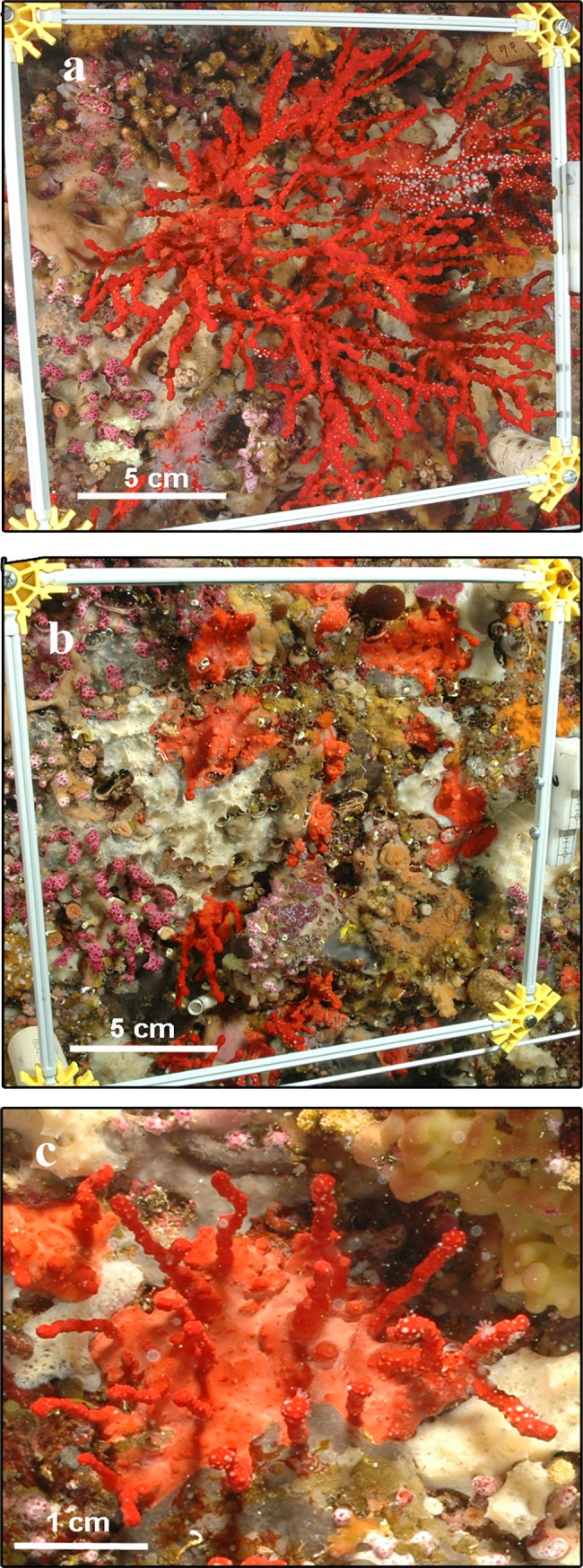
Harvesting effects on red coral populations. Partial mortality at Riou is shown a) before and b) after a harvesting event. c) Detail of re-growth of new branches on a partially harvested colony. Photo credit: Medrecover (www.medrecover.org).

At Maire, permanent plots were installed at 15 m after a harvesting event was observed during June 2002 and monitored till 2009 but due to logistic constraints, pictures were not taken during 2007 and 2008. Colonies that suffered partial mortality were evident because fishermen remove the branches leaving a wide colony basis, short in height with injuries (naked tissue), that unequivocally belongs to an older red coral colony that was recently pruned. In contrast, at Riou the permanent plots were installed in 2005 just before the harvesting event. Between April and June 2006 fishermen were observed in the area and subsequent surveys clearly detected their impact in the permanent plots (Fig. 1).

Our study did not involve any sampling of or damage to red coral colonies. The authors had all the permits provided by the French authority Affaire Maritimes to perform scientific surveys in the study area.

### 3 Demographic parameters

From the photographic series, the harvested and non-harvested colonies were individually identified and compared year to year to estimate mortality rates, number of branches and colony height. We also estimated adult population density (colonies number per 400 cm^2^) and recruitment rates. We considered a recruit the new button like colonies observed ranging from a minimum detection size of 2 mm to 10 mm in height. Annual recruitment rates were estimated as the number of recruits appearing for the first time in a photoquadrat and post-recruitment mortality were estimated by following these recruits through the study period.

With this dataset we were able to assess:

3.1 Changes in size-structure

To assess population size-structure changes and the recovery process, we measured maximum height of all colonies and classified them into five size classes (0–30mm; 30–60mm; 60–90mm; 90–120mm, >120mm). Riou population was measured before harvesting (2005), right after (2006) and four years after (2009). Maire population was measured right after harvesting (2002) and seven years after (2009). Biomass variations were estimated by applying a height-weight polynomial equation to height data. The equation was previously calculated from height and weight data measured on 300 dead red coral colonies collected in previous studies in the same region [35], and from different poaching events [26]. The resulting curve was: Weight (g) = 0.001(Height, mm)^2^ + 0.096(Height, mm)—4.010 (R² = 0.868, P < 0.001) (S1 Fig.).

3.2 Recovery mechanisms

We quantified the cumulative survival probability of non-harvested adults, harvested adults, and new recruits based on annual mortality rates, calculated as follows:

$$m=\frac{\left(1-\frac{N_{t}}{N_{0}}\right)}{\Delta_{t}}$$ (1)

where *m* is the annual mortality rate of *C*. *rubrum* colonies, N is the total number of colonies, and ∆t is the duration of the study period. In addition we assessed the degree of recovery of affected colonies by quantifying the frequency of colonies showing new branches and the number of branches per colony during the study period.

### 4 Simulations of recovery process under different harvesting practices

To analyze whether recovery rates depend on harvesting practices in the long-term, we developed a set of basic stochastic demographic models under two different scenarios with contrasting levels of total (TM) and partial mortality (PM): a) removal of almost all colonies causing 90% TM and 10% PM; b) leaving the basal section in almost all colonies causing 10% TM and 90% PM. The second scenario was based on the observed mortality values at Riou (see results). The two scenarios were calculated by setting initial population densities according to the corresponding levels of total mortality. To run the model we used demographic parameters (adult density, annual recruitment rates, adult and post-recruitment mortality, and biomass increase rates per colony) obtained from the two monitored populations during the study period. The two harvesting scenarios were projected by running 100 stochastic simulations during 30 years according to the following steps:

First we estimated the changes in the number of colonies following the next formulation:

$$\begin{array}{cc} N_{T\left(t\right)}= & N_{R\left(t\right)}+N_{A\left(t\right)}\\ N_{R\left(t\right)}= & N_{R\left(t-1\right)}\left(1-g\right)\left(1-m_{g}\right)+r\\ N_{A\left(t\right)}= & N_{A\left(t-1\right)}\left(1-m_{A}\right)+N_{R\left(t-1\right)}g\left(1-m_{R}\right) \end{array}$$ (2)

where *N*_*T*_ is total population, *N*_*R*_ is total number of recruits, *N*_*A*_ is the total number of adults, *m*_*R*_ and *m*_*A*_ are annual mortality rate of recruits and adults respectively, *r* is the annual recruitment rate and *g* is the proportion of recruits growing to the adult stage:

$$g=\frac{G}{h_{\max}-h_{\min}}$$ 3

where *G* is the recruit growth rate (2.5 mm · yr^-1^ in height, from reference [34]), *h*_min_ and *h*_max_ are the lower and upper height intervals for recruits.

Secondly, we estimated the evolution of biomass recovery using the biomass increases (*ΔB*) per colony and abundance values from the demographic models previously described. Mean (SD) biomass increases were estimated according to the next equations applied to colonies suffering partial mortality at Riou (n = 57) and Maire (n = 67) right after harvesting and 4 and 7 years after respectively:

$$\Delta B=\frac{1}{N}\sum_{i=1}^{N}{\left(\frac{B_{f}-B_{0}}{\Delta_{t}}\right)}_{i}$$ (4)

$$B_{\left(t\right)}={\mathrm{NT}}_{\left(t\right)}.\Delta B$$ (5)

where *B*_*0*_ is the colony biomass right after harvesting, B_f_ is the total biomass at the end of the study period, ∆t is the number of years, and *NT* is the total number of colonies in each population.

## Results

### 1 Harvesting effects

**Fig 2:**
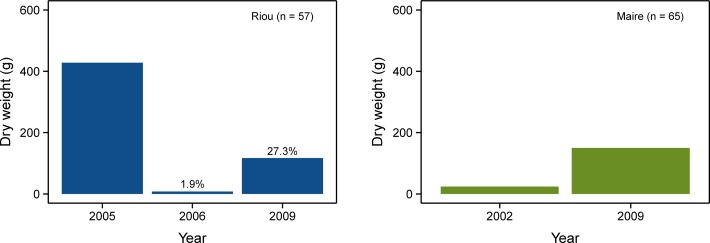
Biomass changes during the study period at (a) Riou and (b) Maire.

**Fig 3:**
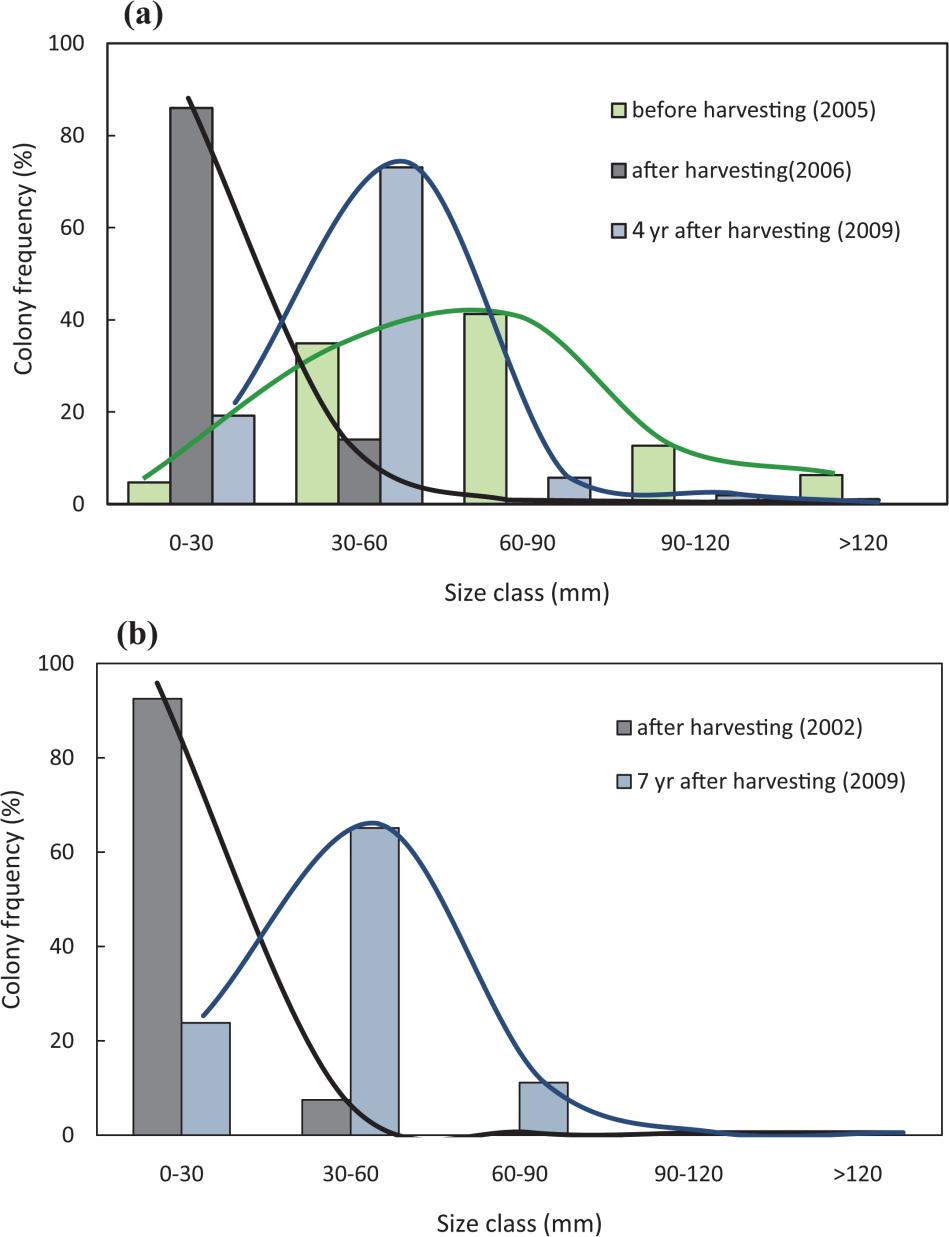
Size-distribution changes on harvested red coral populations. a) Size-distribution at Riou before harvesting, after harvesting, and four years after harvesting. b) Size-distribution at Maire after harvesting and seven years after harvesting.

Harvesting affected 30.1% of the colonies at Riou (n = 209) and 49.6% of the colonies at Maire (n = 139). At Riou, total mortality was around 9.5% and partial mortality was 90.5%, causing a large biomass loss with a 98.5% of reduction in total dry weight (Fig. 2). Harvesting targeted mainly medium and large colonies, triggering a dramatic size-distribution shift toward populations characterized by a large proportion of small colonies (Figs. 1 and 3A). At Maire, because the monitoring started after the harvesting event, we could not determine the loss of biomass nor the size-class distribution shift. Nonetheless, the same size-distribution pattern was observed after the harvesting event with no large colonies present and a great proportion of small-size colonies (Fig. 3B).

### 2 Temporal trends

**Fig 4:**
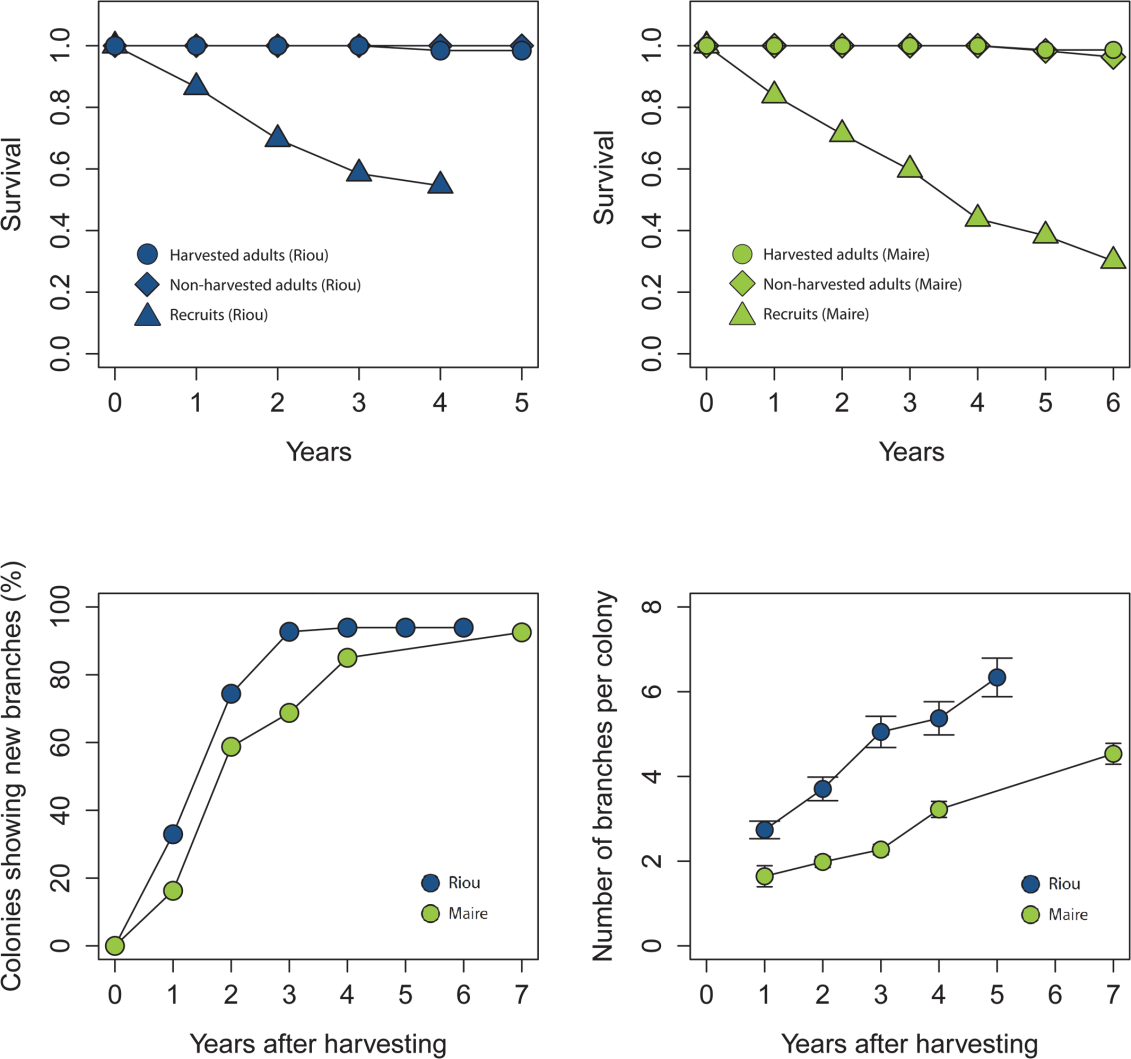
Demographic parameters estimated during the study period at Maire and Riou populations. Cumulative survival probability estimated at Riou (a) and Maire (b) for non-harvested colonies, harvested colonies, and recruits. (c) Temporal variation on the proportion of colonies showing new branches after fishing. (d) Temporal variation of mean (± SD) number of branches after harvesting.

Demographic parameters did not show large differences among sites. Mean annual density was 12.26 ± 0.80 colonies · 400 cm^-2^ at Riou (n = 30) and 11.36 ± 0.52 colonies · 400 cm^-2^ at Maire (n = 30). Densities were quite stable during the study period (S2 Fig.), and recruitment rates were low at both sites: 0.98 ± 1.40 (SD) colonies · 400 cm^-2^ at Maire and 0.21± 0.53 (SD) colonies · 400 cm^-2^ · yr^-1^ at Riou. Harvested colonies suffering from severe partial mortality did not show a differential "post affection" survival as they showed similar high values than unaffected colonies (Fig. 4A, 4B). This could explain the observed stability in adult density in both populations throughout the study period (S2 Fig.). Conversely, survival of recruits was very low and decreased rapidly at both populations (Fig. 4A and 4B).

### 3 Rate of recovery

The two studied red coral populations demonstrated very low rate of recovery. A small increase of biomass was observed at Riou after four years, representing 22% of the initial biomass recorded prior to the harvesting event (Fig. 2). A slight recovery was also observed by comparing size-class distribution from Riou and Maire populations after four and seven years respectively (Fig. 3). Despite a general increase in the proportion of medium size-classes, there was no recovery of the largest sizes (>100mm) and very little of colonies >80mm at Riou. At the end of the study, small colonies (0–60 mm) still represented a large proportion of the whole population (92% at Riou and 89% at Maire). Re-growth was observed in more than 75% of the harvested colonies after two years at Riou and four years at Maire (Fig. 4C). Mean number of branches in harvested colonies also increased quickly in both populations (Fig. 4D).

### 4 Recovery process simulations

**Fig 5:**
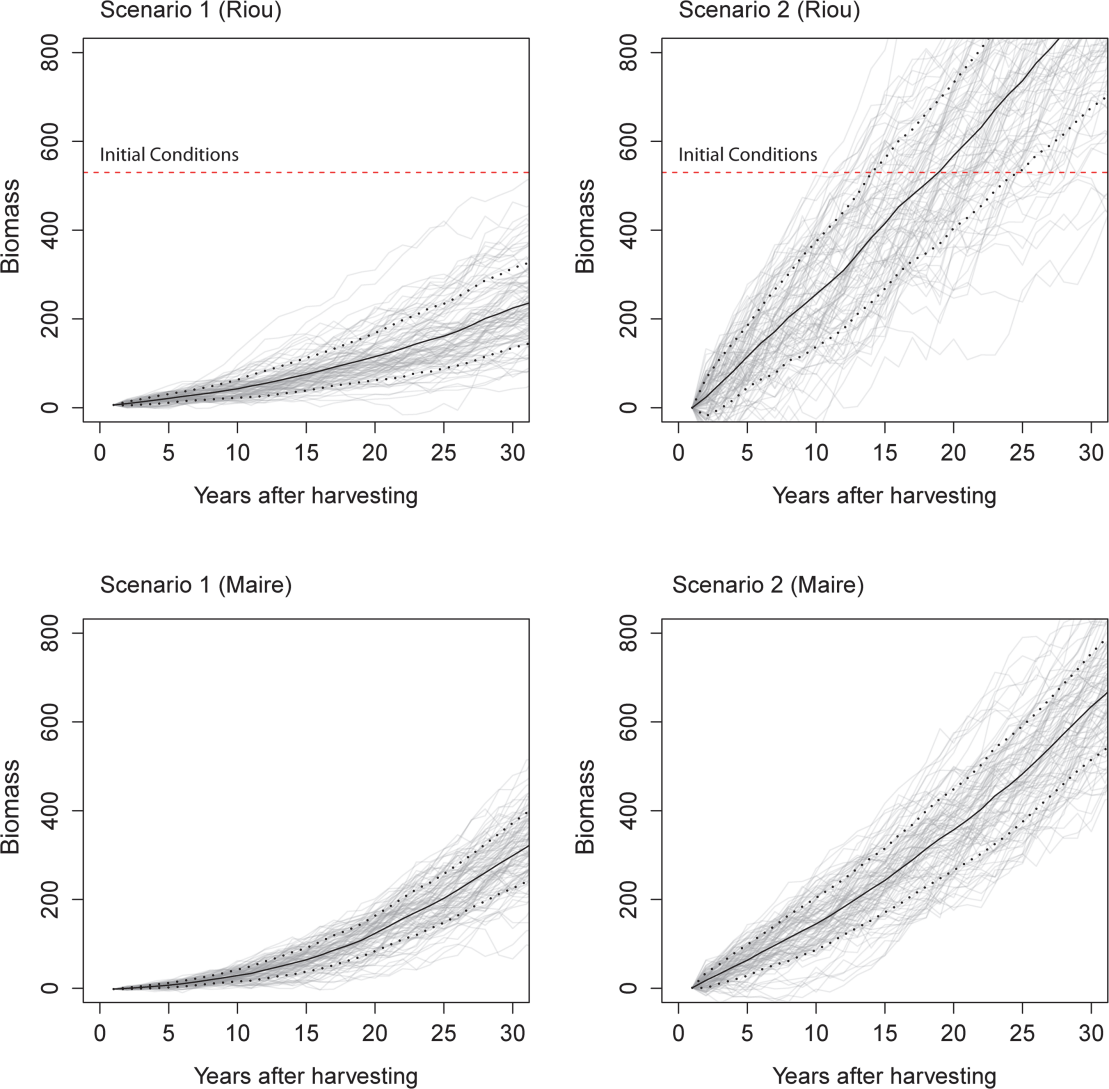
Biomass projections of red coral populations to compare different harvesting scenarios. Scenario 1) 90% total mortality and 10% partial mortality. Scenario 2) 10% total mortality and 90% partial mortality. Horizontal broken lines show initial conditions at Riou. Black lines represent the mean trend and dot lines the standard deviation (n = 100).

Biomass simulations based on demographic projections showed that harvesting practices strongly influence the recovery process of *C*. *rubrum* populations. Harvesting practices in which the colony basis remains attached showed recovery times from 15 to 25 yr, whereas events causing total mortality (eradicating the whole colony) slowed down the recovery process, preventing the populations from recovering to initial conditions even 30 years after being harvested (Fig. 5). Riou population showed a slightly higher recovery rate than Maire population. However the influence of harvesting practices at slowing down the recovery rate when increasing total mortality was consistent for both populations (Fig. 5).

## Discussion

This study provides new insights into the recovery mechanisms of a heavily exploited precious coral and illustrates how different harvesting practices can strongly modulate the recovery of affected populations. Our results also highlighted the detrimental effects of harvesting and the low rate of recovery displayed by these long-lived invertebrates.

Harvesting heavily impacted red coral populations has led to drastic reductions of biomass and size-structure shifts towards populations dominated by small colonies. Although both populations still persisted at the end of the study period (after 4 and 7 years), they were far from initial conditions in terms of biomass and sizes. The slow recovery processes reported here confirms the long-lasting negative consequences of harvesting on slow-growing sessile organisms and indicates that extractive practices under the current legal framework are not sustainable from a long-term perspective [5].

We investigated the relative contribution of reproduction and re-growth of harvested colonies as drivers of recovery of red coral populations. The high survival of harvested colonies suffering partial mortality and fast growth of new branches demonstrated that re-growth plays a key role in recovery and persistence of *C*. *rubrum* (Fig. 4C, 4D). Similar to previous long-term studies conducted in French and Spanish localities [13], [26], the observed low recruitment rates and very low survival probability of new recruits suggest that recovery through reproduction is very limited (Fig. 4A, 4B). This demographic strategy is shared by other temperate gorgonian [36], and is concordant with life-history theory, which states that adult survival is inversely proportional to reproductive success [37]. Indeed, biomass simulations developed to compare different harvesting practices showed that when the whole colony is removed and thus reproduction is the main recovery mechanism, the process is much slower and it can take much more than 30 years to return to initial conditions pervious to harvesting. Conversely, when fishermen leave the basal section of the colonies recovery is enhanced through re-growth of new branches (Fig. 5). The biological reference point used in this study (size-class distributions at Riou prior to harvesting) does not represent an undisturbed population; therefore, recovery leading to pristine conditions would take longer periods than those reported here. Further, we must note that the models used here do not completely account for the complexity of demographic processes in long lived species. *C*. *rubrum* is characterized by a low dispersal that leads its populations to be generally self-seeding [27], [38], and breeding units seem also to be restricted in space, suggesting that density may play an important role in the reproduction of this species [28]. Thus, events causing total mortality on adult colonies could lower the potential for recovery even more through reproduction [40]. Alternatively, negative density-dependent processes affecting recruitment and post-recruitment survival were also reported in corals due to intra-specific competition and resources limitation (i. e. availability of suitable substrate to settle) [39]. Further research should therefore address these questions by applying more complex modeling techniques to long-term experimental data. However, the observed large and consistent differences on population trajectories depending on harvesting procedures revealed by our study represent an important first step in the characterization of the ecological consequences of different harvesting practices on *C*. *rubrum* populations.

Reported recent declines on the Mediterranean yields sparked a wide debate about the conservation status of *C*. *rubrum*. This discussion raised questions about the sustainability of this fishery and if this species should be categorized as threatened [7], [19], [20], [21], [41]. Our results show that populations demonstrate a high degree of persistence in terms of density due to high survival of affected colonies. This may explain the existence of red coral populations despite intensive harvesting during the past few centuries. Nevertheless, we emphasize the large reduction of biomass and the size-distribution shifts toward populations dominated by small colonies likely hindered their structural function. A general simplification of benthic communities may also have negative consequences for multiple trophic levels that use these habitats as shelter during early life-stages [10], [42]. This finding should be especially relevant for policymakers. Indeed, abundance is a parameter commonly used by international organizations such as IUCN or CITES to categorize vulnerability levels in commercial species [7], [43]. However, our results challenge the suitability of abundance-based metrics to assess the conservation status of clonal (modular) organisms by showing that large declines in biomass due to partial mortality of colonies can remain masked. Furthermore, abundance measures do not reflect the reduction in reproductive potential caused by harvesting colonial organisms, which exhibit a size-based exponential increase in reproductive output [44]. The development of a new metric focused on size and biomass parameters is crucial to assess the conservation status of precious corals and to develop sustainable fisheries management plans.

On the other hand, benthic communities are currently facing multiple perturbations derived from the ongoing global change such as warming and ocean acidification [16], [17], [18], [45]. The precautionary principle accounting for potential synergistic effects of fishing and climate warming should thus be considered in these new regulations [15], [16], [46]. While climatic perturbations are generally diffuse and difficult to minimize, fishing restrictions on no-take areas can be useful tools at a local scale to enhance the resilience of sessile invertebrates through increased larval production of large individuals [47]. MPAs also enhance the recovery of exploited populations in terms of biomass and size-structure [14], [26]. However given the low recruitment rates and limited dispersal of gorgonian larvae [27], it is unlikely that protected populations act as a source to exploited populations ensuring their long-term persistence [28]. Further, poaching and diving activities hinder the enhancement of coral populations’ recovery within MPAs when regulations are not enforced [27]. Therefore, we should not just consider improving the actual network of MPAs but also moving towards a more restrictive legal framework outside of the protected areas. Several new regulations have been proposed during the last decades to improve *C*. *rubrum* fisheries, including the establishment of annual quotas and minimum harvesting sizes and the ban on using dredges. However, these current guidelines are still largely based on untested assumptions and have failed to improve the sustainability of coral fisheries [20]. For instance, a significant step was the recent recommendation on total ban on harvesting of *C*. *rubrum* populations under 50m depth [22], although poaching in shallow waters is still widespread and individual countries may avoid this prohibition by developing specific management plans. Rotating systems were also proposed for precious corals fisheries in the Pacific and the Mediterranean though there still is a large uncertainty around the recovery periods of affected populations [20]. Here, we show how red coral populations may take much more than three decades to recover and that total removal of colonies can significantly slow down this process further. Our approximations are consistent with previous studies conducted within French and Spanish MPAs where populations showed only partial recovery after 20 to 30 yrs of protection [11], [14], [26]. Additionally, there has been a recent development of new jewelry manufacturing processes that allow using small pieces of coral that can be ground to powder and mixed with epoxy or other substances [22]. This development worsens the effects of harvesting by targeting whole size ranges and including entire colonies. Recovery of populations affected by these new practices might be even more jeopardized since re-growth mechanisms are dramatically limited. Finally, this study highlights the importance of long-term ecological monitoring programs because they provide reliable data on key ecological processes such as the recovery patterns of natural populations after human-induced disturbances and enable the improvement of conservation strategies.

## Supporting Information

S1 Fig Height – weight relationship obtained from death corals (n = 300). (TIF)

S2 Fig Annual adult densities at the study sites. (TIF)
