# Supplementary figures and images for: Harvesting Effects, Recovery Mechanisms, and Management Strategies for a Long-Lived and Structural Precious Coral
Source: PLoS One. 2015 Feb 23;10(2):e0117250. doi: 10.1371/journal.pone.0117250 (PMC4337904; doi:10.1371/journal.pone.0117250)

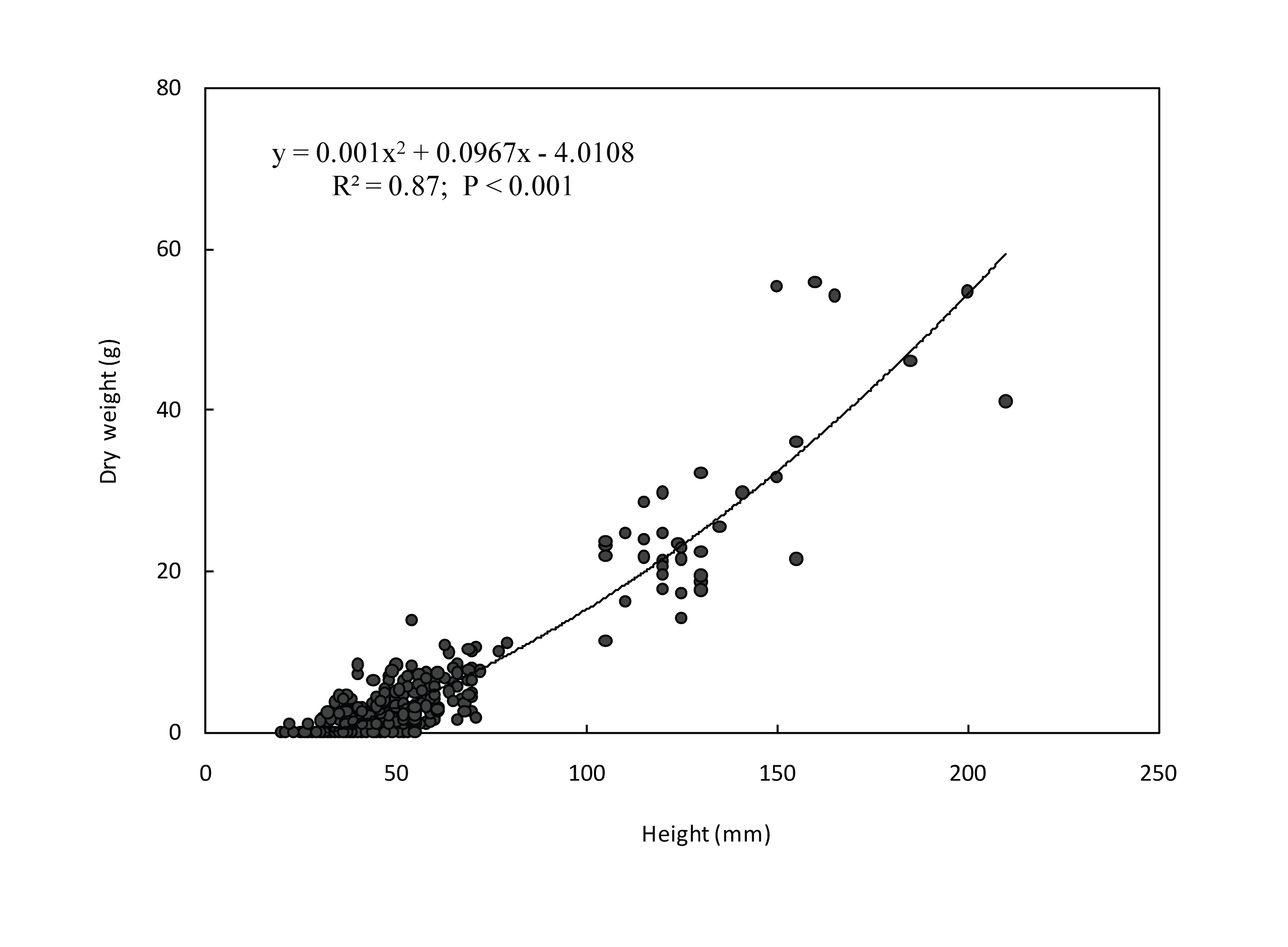

Supplement: S1 Fig — (TIF) [file pone.0117250.s001.tif]

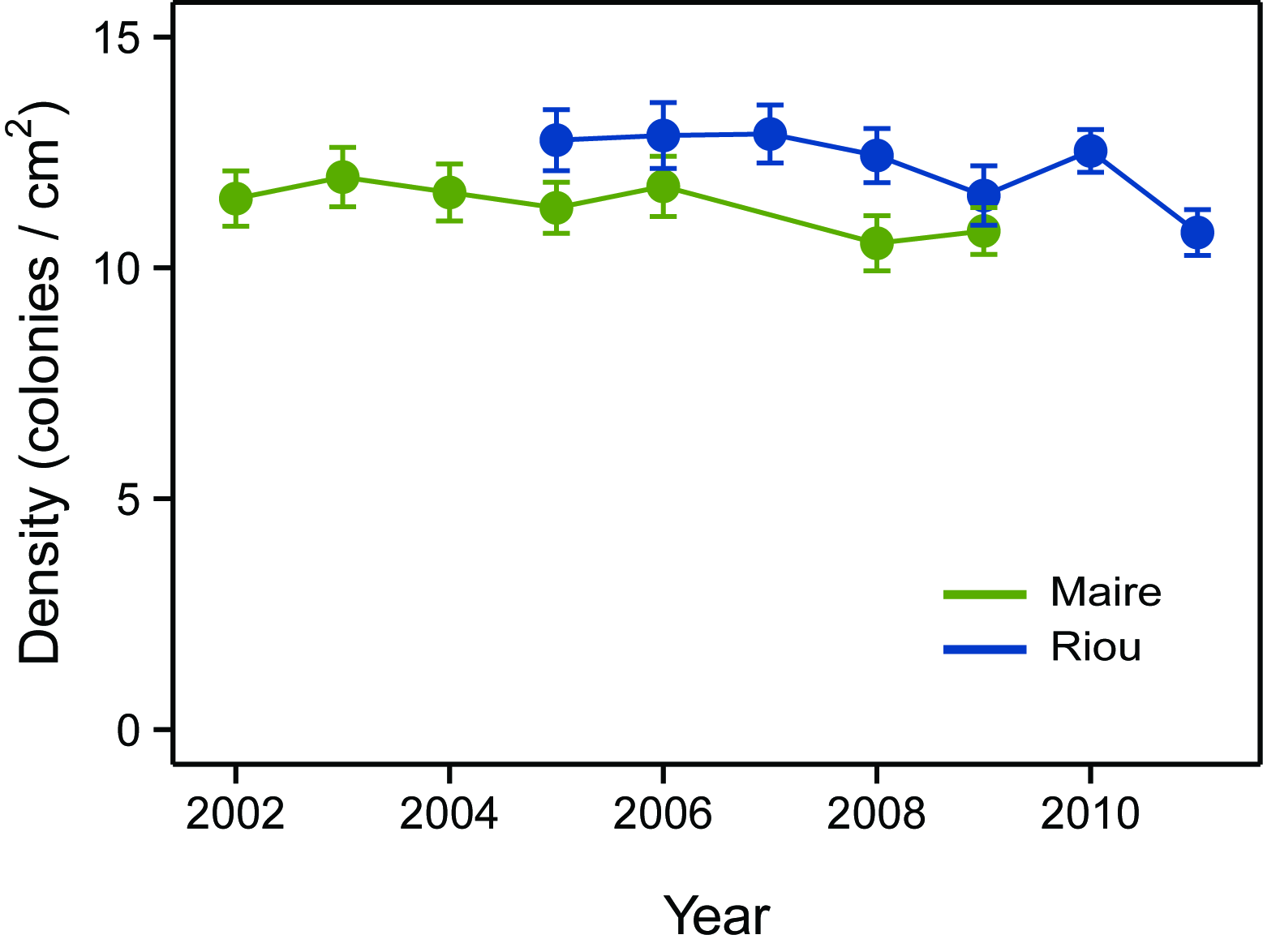

Supplement: S2 Fig — (TIF) [file pone.0117250.s002.tif]
